# Optimizing the IPSA Conditions to Improve the Treatment Plan Quality in Brachytherapy for Cervical Cancer

**DOI:** 10.1155/2022/6499744

**Published:** 2022-03-12

**Authors:** Xinglong Yang, Zhouyu Li, Zhantuo Cai, Xi Tang, Jinquan Liu, Shuzhong Cui, Mingyi Li

**Affiliations:** ^1^Department of Radiation Oncology, Affiliated Cancer Hospital and Institute of Guangzhou Medical University, Guangzhou 510095, China; ^2^Affiliated Dongguan People's Hospital, Southern Medical University, (Dongguan People's Hospital) Radiotherapy Ward 3, Dongguan 523059, China; ^3^Department of Abdominal Surgery, Affiliated Cancer Hospital of Guangzhou Medical University, Guangzhou 510095, China

## Abstract

Recent prevalent use of three-dimensional image-guided brachytherapy (3D brachytherapy) has dramatically improved the treatment outcomes of cervical cancer. Inverse planning simulated annealing (IPSA) is one of the commonly used algorithms in 3D brachytherapy, but different conditions may affect the treatment plan quality. In this study, we compared HRCTV (high-risk clinical target volume) D90 (dose prescription) and HRCTV D95 D2cc (dose received by 2.0cc) of the rectum, bladder, and sigmoid in 30 patients with cervical cancer under four IPSA conditions. The HRCTV D90 (mean ± SD cGy) was 607.32 ± 37.86, 599.01 ± 23.62, 598.67 ± 13.07, and 596.45 ± 10.94 in four groups, respectively. The HRCTV D95 was 558.19 ± 38.51, 558.17 ± 25.72, 557.03 ± 16.12, and 555.26 ± 12.78, respectively. The sigmoid D2cc was 282.96 ± 44.84, 273.14 ± 60.69, 268.94 ± 62.32, and 292.69 ± 52.44. HRCTV D90, HRCTV D95, and sigmoid D2cc were not statistically different among the four groups (*p* > 0.05). However, the target fitness in group one, especially at the cervix, was poor. The rectum D2cc was 351.49 ± 32.90, 361.49 ± 28.09, 370.82 ± 24.44, and 375.33 ± 30.90. The rectum D2cc in group one was the lower than that in group three and group four (*p* < 0.05). The bladder D2cc was 423.59 ± 31.39, 380.75 ± 37.25, 383.27 ± 32.55, and 385.22 ± 25.79. The bladder D2cc in group one was higher than the other groups (*p* < 0.05). The maximum rectum limit dose (400cGy) is lower than the bladder (500cGy), and HRCTV is a whole in the IPSA algorithm; these result in the insufficiency or even absence of cervix dose that first need to meet in clinics. In conclusion, IPSA condition optimization can improve the quality of treatment plan in 3D brachytherapy and make it closer to clinical practice.

## 1. Introduction

Cervical cancer is the fourth common cancers in women after breast cancer, colorectal cancer, and lung cancer, seriously threatening women's health worldwide. According to the latest global cancer statistics, the number of new cases of cervical cancer was 604000 and the number of deaths was 342000 in 2020 [1]. In recent years, the incidence of cervical cancer is decreased due to screening, economic development, reduced risk of persistent HPV infection, improved hygiene, low fertility rate, and reduction in sexually transmitted diseases [2]. The main treatment methods for cervical cancer are radical radiotherapy and cisplatin-based chemotherapy. Complete radical radiotherapy including pelvic external-beam radiotherapy (EBRT) and brachytherapy plays a critical role in the treatment of cervical cancer [3]. Brachytherapy, as a supplement to EBRT, plays an important role in the treatment of cervical cancer. It is developed from the traditional intracavitary brachytherapy (2D brachytherapy) to the present three-dimensional image-guided intracavitary and interstitial brachytherapy (3D brachytherapy) [4, 5]. 2D brachytherapy is to prescribe a radiation dose to an empirical point and does not necessarily reflect the actual dose to the tumor [6]. 3D brachytherapy is based on CT or MRI scan images, and the dose can be visualized and adjusted on the clinical target and the surrounding organs at risk (OARs) [7, 8]. 3D brachytherapy can improve the tumor treatment dose and reduce the side effects which are increasingly accepted by hospitals. 3D brachytherapy has two common methods for making the plan: graphical optimization (GO) and inverse planning simulated annealing (IPSA) [9]. In general, IPSA significantly increased target coverage, reduced dose to OARs, and shortened planning time compared to GO. In clinical practice, the conditions of IPSA routinely used by different hospitals and physicists are not uniform and differ greatly [10, 11]. In this study, we compared the influence of four IPSA conditions on 3D brachytherapy plan, promoting the more rational application of IPSA in clinics.

## 2. Materials and Methods

1. Clinical data: in this study, 30 patients with pathologically confirmed cervical cancer (with stage IIA1-IVB) were selected. All patients were given external irradiation with a dose of 45–50.4 Gy/25–28 F/5–5.5 W, followed by 3D brachytherapy, 1-2 times/week, with a dose of 6 Gy*∗*5F. The general characteristics of patients are given in Table 1.
2. 3D brachytherapy process and CT image collection: patients empty the rectum and take the lithotomy position in a gynecological bed. The perineum and the peripheral 15 cm area were disinfected three times with iodophor disinfectant. After urethral orifice was disinfected, a catheter was inserted. The depth and curvature of the intrauterine tube were determined according to the uterine position of patients. The number, position, and depth of the insertion needles were determined in combination with the patient's tumor extent and the location of adjacent organs. Following the intrauterine tube and the needle insertion, the catheter was fixed, and then, 100–250 ml normal saline was dripped into the bladder. CT scan was performed for positioning, ranging from the upper edge of the four lumbar vertebrae to 5 cm below the ischial tuberosity, with a thickness of 2.5 mm. CT images were sent to the brachytherapy treatment planning system (Oncentra Brachy V4.3) for contouring of HRCTV and OARs.
3. Contouring of HRCTV and OARs: we used the MRI enhanced scan images after external irradiation and reference to the guidance of GEC-ESTRO (The Groupe Européen de Curiethérapie (GEC) and the European Society for Radiotherapy and Oncology (ESTRO)) [7, 9]. The HRCTV was contoured from the cervix to the uterine and the vaginal end according to the pelvic MR results and the gynecological examination before and after external irradiation. The OARs are then outlined in turn: rectum, bladder, and sigmoid. The doses of OARs are limited according to guideline [12].
4. IPSA plan conditions: the selected patients` brachytherapy treatment plans were designed with four different IPSA conditions. The first group used routine parameter conditions (Table 2), the second group used optimization parameter conditions (Table 3), the third group received optimization parameter conditions combined with clinical practice manual adjustment, and the fourth group used optimization parameter conditions combined with target optimization (Table 4).
5. Observation subjects: after completing IPSA plans under different parameter conditions, HRCTV D90 and HRCTV D95 were recorded according to the DVH diagram. In this study, the dosiology of OARs (such as the rectum, bladder, and sigmoid) concerned in the 3D brachytherapy plan were monitored, and D2cc is closely related to adverse reactions of normal tissues [13].
6. Statistical analysis: SPSS 26.0 was used for statistical analysis. The intergroup *t-*test was used for dosimetry comparison of HRCTV and OARS. *P* < 0.05 was considered as a significant difference between the two groups.

## 3. Results

### 3.1. Dosimetry Comparison of the Four Groups

HRCTV D90, HRCTV D95, and D2cc doses of OARs such as the rectum, bladder, and sigmoid were collected for comparative analysis according to DVH plots.

**Figure 1:**
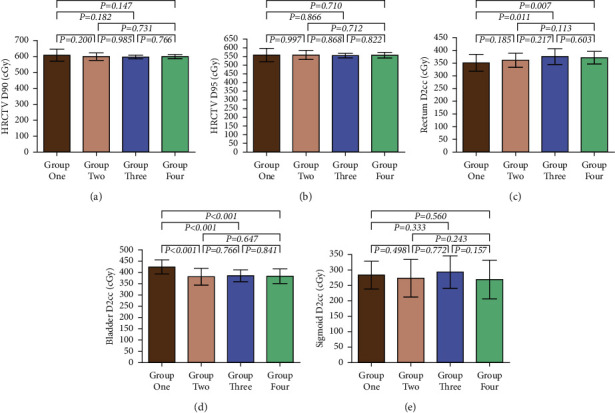
Radiotherapy dose comparison in four groups.

Figure 1(a) shows the comparison result of HRCTV D90 of the four groups. The HRCTV D90 dose of group one was the highest among the four groups. There was no statistical difference among the other three groups (*p* > 0.05).

Figure 1(b) shows the comparative analysis of HRCTV D95 of the four groups. The HRCTV D95 dose of group one was higher than those of the other three groups. There was no statistical difference among the other three groups (*p* > 0.05).

Figure 1(c) shows the result of D2cc dose of the rectum. The D2cc dose of the rectum in group four was the highest, and the dose in group one was the lowest. The D2cc dose of the rectum in group one was lower than that in group three (*p*=0.011) and group four (*p*=0.007). There was no statistical difference among the other groups (*p* > 0.05).

Figure 1(d) shows the comparative analysis of the bladder D2cc doses of the four groups. The average dose of group two was the lowest among the groups. The dose of group one was the highest among the four groups. There was a statistical difference between the dose of group one and the other three groups (*p* > 0.001). However, there was no statistical difference among the other three groups (*p* > 0.05).

Figure 1(e) shows the comparative analysis of D2cc dose of sigmoid among the four groups. Group two had the lowest dose of sigmoid D2cc, and group four had the highest dose among the four groups. However, there was no statistical difference in the D2cc dose of sigmoid among all groups (*p* > 0.05).

Table 5 provides the average dose values of dosimetry including HRCTV D90, HRCTV D95, and D2cc doses of OARs including the rectum, bladder, and sigmoid of the patients.

### 3.2. Comparison of Dosimetry Distribution in the Four Groups

The above results of dosimetry analysis showed that group one had the highest dose of bladder exposure and the lowest dose of rectum exposure among the four groups. We further analyzed the dose distribution of target region in different cross-sectional sections and the cross-sectional sections of uterine segment (the upper quarter of HRCTV), upper cervical segment (the upper middle quarter of HRCTV), cervical segment (the lower middle quarter of HRCTV), and vaginal segment (the lower quarter of HRCTV).

**Figure 2:**
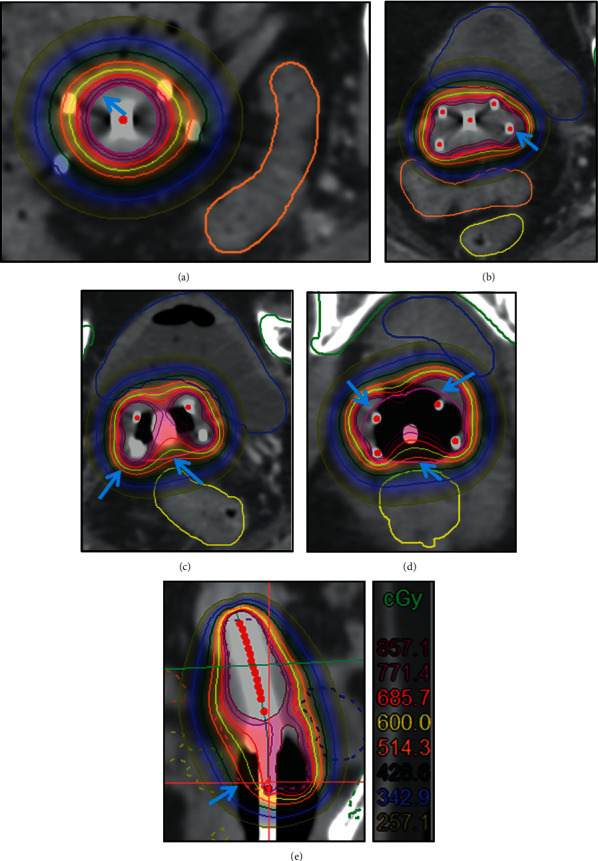
Radiotherapy dose curve distribution in different cross-sections of group one. (a) Uterine segment. (b) Upper cervical segment. (c) Cervical segment. (d) Vaginal segment. (e) Sagittal plane. The blue arrow indicates improper dose coverage (yellow ring is the 600 cGy line, and red ring is the HRCTV line).

Figure 2 shows the distribution of dose curve at each cross-section in uterine segment, upper cervical segment, cervical segment, and vaginal segment, as well as the sagittal plane of group one. We found that the dose curve shifted toward the uterine segment and posterior wall of the bladder.  Figure 2(a) shows that the 600 cGy (yellow) dose line in the uterine segment had exceeded the target region outlined (red). Figures 2(b) and 2(c) show the 600 cGy dose line did not have complete coverage in the upper cervix segment and cervix segment, especially, the dose was not enough in the region of rectal anterior wall.  Figure 2(d) shows the 600 cGy dose line exceeded the target region at the anterior vaginal wall and lacked at the posterior vaginal wall. This indicated that the dose in the posterior wall of the bladder was too high and the dose in the posterior vaginal wall was not sufficient.  Figure 2(e) shows the overall deviation of the dose line toward the upper part of the uterine segment and the posterior wall of the bladder.

**Figure 3:**
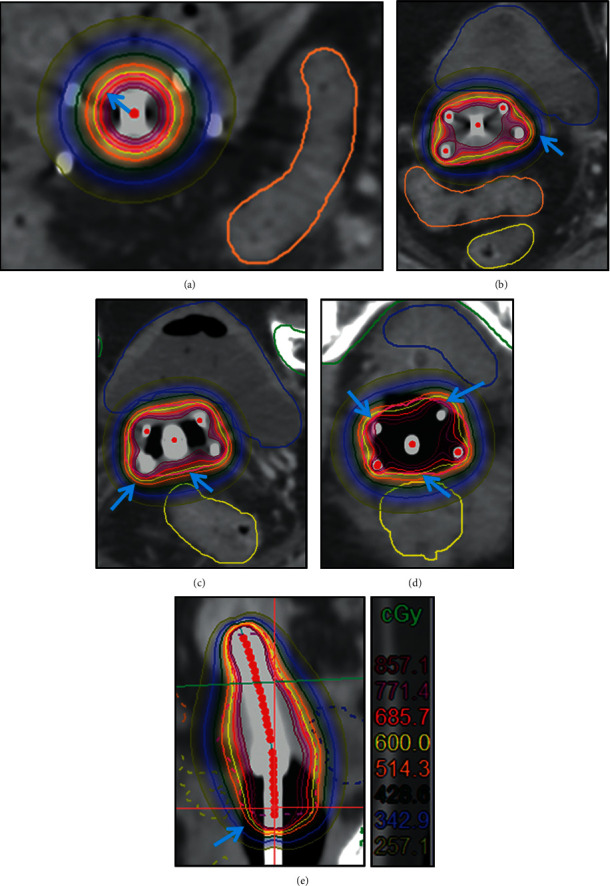
Radiotherapy dose curve distribution in different cross-sections of group two. (a) Uterine segment. (b) Upper cervical segment. (c) Cervical segment. (d) Vaginal segment. (e) Sagittal plane. The blue arrow indicates improper dose coverage in group one was improved in group two (yellow ring is the 600 cGy line, and red ring is the HRCTV line).

Figure 3 shows the distribution of dose curve at each cross-section in uterine segment, upper cervical segment, cervical segment, vaginal segment, and the sagittal plane of group two. We found that the dose curve covering the target region in group two is better than in group one.  Figure 3(a) shows that the 600 cGy dose line in uterine segment is closer to the target outlines in group two than group one. Figures 3(b) and 3(c) show the 600 cGy dose line in the upper cervical segment and cervical segment HRCTV coverage is improved in group two compared to group one.  Figure 3(d) shows that the 600 cGy dose line coverage at the vaginal wall in group two is significantly better than that in group one.  Figure 3(e) shows the target coverage of 600 cGy dose line in group two is significantly better than that in group one.

**Figure 4:**
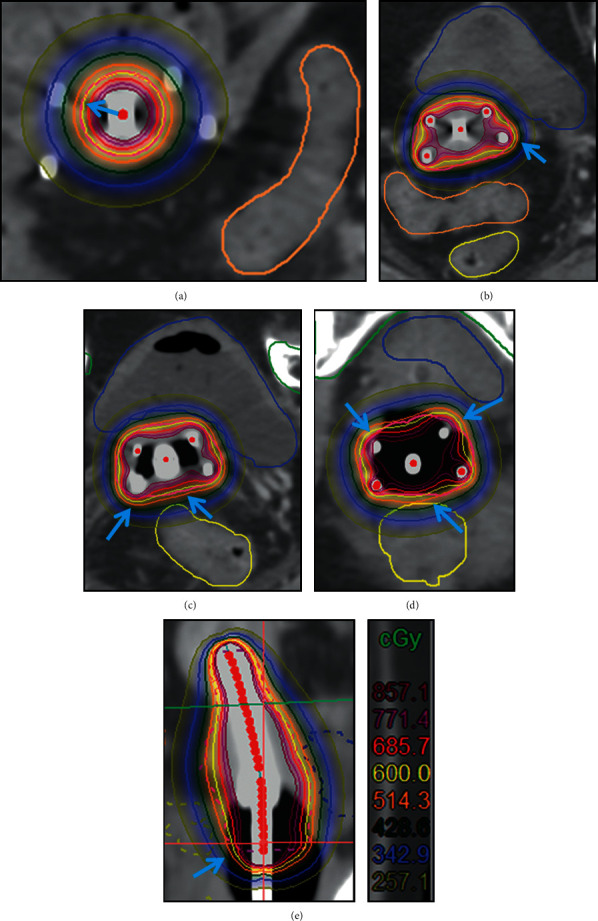
Radiotherapy dose curve distribution in different cross-sections of group three. (a) Uterine segment. (b) Upper cervical segment. (c) Cervical segment. (d) Vaginal segmen. (e) Sagittal plane. The blue arrow indicates improper dose coverage in group one was improved in group three (yellow ring is the 600 cGy line, and red ring is the HRCTV line).

Figure 4 shows the distribution of dose curve at each cross-section in uterine segment, upper cervical segment, cervical segment, vaginal segment, and the sagittal plane of group three. Group three used the same conditions as group two and combined with the actual situation of patient (for example, in the case there were invasions of uterine segment, bladder posterior wall, or rectum anterior wall, it is necessary to manually adjust the boost dose in the invasive region). Figures 4(a)–4(d) show that the dose lines at uterine segment, upper cervical segment, cervical segment, and vaginal segment had no significant change compared with group two.  Figure 4(e) shows that the dose curve line targeted overall coverage in group three is further optimized compared with group two.

**Figure 5:**
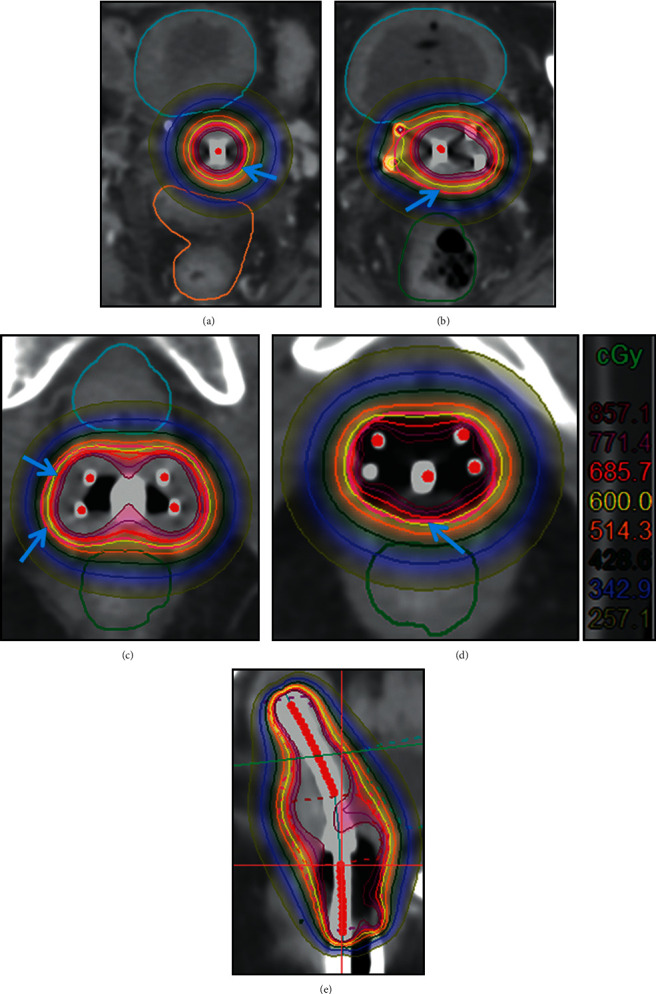
Radiotherapy dose curve distribution in different cross-sections of group four. (a) Uterine segment. (b) Upper cervical segment. (c) Cervical segment. (d) Vaginal segment. (e) Sagittal plane. The blue arrow indicates improper dose coverage in group one was improved in group four (yellow ring is the 600 cGy line, and red ring is the HRCTV line).

Figure 5 shows the distribution of dose curve at each cross-section in uterine segment, upper cervical segment, cervical segment, vaginal segment, and the sagittal plane of group four. Group four used the same conditions as group two and introduced ultra high-risk clinical target volume (UHRCTV, it mainly refers to the lower middle quarter of HRCTV cervical segment; this section in clinical is more needed to be meet the dose than uterine segment, upper cervical segment, and vaginal segment.). In group four conditions, dose coverage first follows the principle of satisfying UHRCTV. In clinical practice, this region needs to be satisfied first. Moreover, because this region is most close to the posterior wall of bladder and the anterior wall of the rectum, it is easily affected. IPSA is not able to differentiate this region from the uterine segment, upper cervical segment, and vaginal segment. Thus, UHRCTV targets were introduced to further define this region to optimize the dose. Figures 5(a)–5(d) show that 600 cGy dose lines in uterine segment, upper cervical segment, cervical segment, and vaginal segment in group four were highly consistent with HRCTV.  Figure 5(e) shows that the dose curve covering the cervical region is further optimized, and the doses in the uterine segment and the vaginal segment are not too high.

### 3.3. Dosimetry Distribution Comparison of the Same Patient under Four Conditions

**Figure 6:**
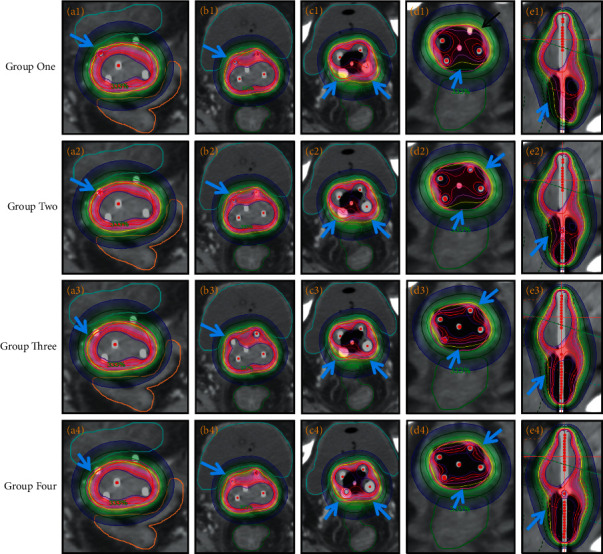
Radiotherapy dose curve distribution in different cross-sections of the same patient. (a) Uterine segment. (b) Upper cervical segment. (c) Cervical segment. (d) Vaginal segment. (e) Sagittal plane. The blue arrow indicates improper dose coverage in group one was improved in other groups (yellow ring is the 600 cGy line, and red ring is the HRCTV line).

Based on the above analysis, IPSA can significantly optimize the distribution of dose curve by limiting the maximum dose on target surface and minimum dose on target volume and further optimize the distribution of dose curve by manually adjusting the actual clinical tumor invasion. Introducing the concept of UHRCTV in the cervical region to further define conditions can further optimize and reduce manual adjustment. Group four was the best (Figure 6), but the introduction of UHRCTV in group four increased the time of delineating target areas. In clinical practice, we mainly use group three and fully combine the actual situation of patients to make reasonable use of IPSA.

## 4. Discussion

According to the global cancer statistics in 2020, the new cases and deaths of cervical cancer increased to 604000 and 342000, respectively [1]. The treatment of cervical cancer is mainly radiotherapy, and it is suitable for patients at all stages. According to the guidelines of the National Comprehensive Cancer Network (NCCN, 2019 edition) and the International Federation of Gynecology and Obstetrics (FIGO, 2018 edition), radical radiotherapy and concurrent platinum-containing chemotherapy are the preferred treatment modalities for locally advanced cervical cancer patients with stages IB3, IIA2, IIB, and above. Surgery is recommended for patients with early stages except for those who require fertility sparing [12]. For patients with early stage cervical cancer (such as IA, IB1, IB2, and IIA1) and nonfertility sparing requirement, the primary treatment is surgery or radical radiotherapy and (or) concurrent platinum-containing chemotherapy; both treatments are equally effective. Radical radiotherapy for cervical cancer includes two parts: EBRT and brachytherapy. With the development of radiotherapy technology, the survival time and quality of life of patients with cervical cancer have been significantly improved [14]. 3D brachytherapy, in particular, is an integral part of radical radiotherapy for cervical cancer. Its advantages include that it provides high dose of radiation in target areas for a radical cure of cancer and ensure low dose in surrounding organs [6, 8]. The gradient descent characteristic of brachytherapy can effectively eliminate tumor cells while minimizing radiation side effects on OARs.

With the application of imaging technologies such as CT and magnetic resonance imaging (MRI) in brachytherapy, the dosimetry of 3D brachytherapy is significantly improved compared with traditional 2D brachytherapy [15, 16]. The 3D brachytherapy planning system used in this study was Oncentra Brachy V4.1, which can be designed using manual planning (GO) or IPSA. In clinical practice, we found that manual planning is laborious and requires close cooperation and communication between skilled physicists and clinicians to make optimal planning. In 3D brachytherapy plan design, it is critical to balance radiation source location and radiation duration to ensure sufficient dose in HRCTV and reduce exposure to OARs. IRCTV has been defined and widely mentioned in 3D brachytherapy, but it is not widely used in clinical practice in China, so the influence of IRCTV is not considered in this study. IPSA with the help of computer has powerful algorithms to simultaneously evaluate HRCTV and OARs to optimize the dose distribution. Therefore, IPSA has better result than GO in terms of acceptable HRCTV dose and few exposures to OARs [17]. IPSA plan is better than GO plan as long as the plan limit parameters are properly selected [18].

In this study, four different IPSA plan conditions were used to compare the target dose, the fitness of target dose curve, and the exposure dose to surrounding organs. The average dose of HRCTV D90 and D95 is, respectively, 607.32 ± 37.86 (cGy) and 558.19 ± 38.51 (cGy) in group one, and the HRCTV D90 dose in group one is the highest in four groups. Moreover, the dose distribution is close to the bladder side, uterine segment, and vaginal segment, which is in contrast to the cervical segment dose that should be satisfied first for the brachytherapy of cervical cancer. This was caused by the fact that the surface dose of HRCTV was only limited to 600 cGy when the IPSA plan was designed. We further restricted the conditions and limited the volume dose of HRCTV to 600 cGy, so as to reduce the possibility of 600 cGy line shift to the bladder side, uterine segment, and vaginal segment. After introducing the volume dose limiting parameter of the target area, the fitness of the target area was significantly improved in group two compared with group one. The uterine segment and vaginal segment had better fitness because they were round. The cervical segment was prone to low dose due to their irregularity. This situation is often caused by poor insertion of the needle, abnormal distortion of the anatomical position of cervix, or the HRCTV too close to rectum, bladder, and sigmoid. In clinical practice, appropriate manual adjustment should be carried out according to the clinical situation of patients to obtain the optimal 3D brachytherapy treatment plan.

Group three is added on the basis of group two and combined with clinical manual adjustment to optimize the low dose of cervical segment, so as to further optimize the dose and fitness of target area and the dose of OARs within an acceptable range. Through the comparative analysis of the above three groups, we found that when HRCTV D90 ≥ 600 cGy was met, there was no difference in the four regions of uterine segment, upper cervical segment, cervical segment, and vaginal segment. IPSA did not distinguish cervical segment, while the cervical segment was the key radiotherapy area of the 3D brachytherapy in actual clinical practice, and the target dose in this area could not be sacrificed due to the proximity of the rectum, bladder, and sigmoid. The brachytherapy plan obtained by group four showed that after the introduction of UHRCTV, the dose and fitness of the target area were significantly improved in the cervical segment, and the fitness of the uterine segment and vaginal segment was also significantly improved. The 3D brachytherapy plan obtained by group four was the most consistent with the target dose and target fitness and clinical practice, but it had one more target sketching, which increased the workload of physicians. In the practical application of clinical IPSA, group three is most commonly used as the standard, which can meet the dosage requirements of most cases.

The idea for this study began with an article reporting that the IPSA plan system was significantly superior to GO [10]. GO often requires more skilled physicists and even removed the especial dwell points of radioactive source to optimize the plan [19]. This study initially designed a GO group, but it was abandoned due to the need for skilled 3D brachytherapy plan physicists and cumbersome operation. The practical analysis proves that the IPSA plan system still has obvious advantages. Some of the dwell points removed by the IPSA plan need to be manually filled in some clinical situations, such as the dwell points at the cervix are removed due to some reasons. The purpose of this study is to discuss how to optimize IPSA plans to better serve the clinical conditions; it has certain limitation; we know that better dose distribution depends on the distribution of the uterine tube and the needle. The premise of using the IPSA plan system obtained better treatment plan to have better distribution of the uterine tube and the needle, which needs skilled clinicians in the process of 3D brachytherapy.

In conclusion, in the design process of 3D brachytherapy plan for cervical cancer, we need to select the appropriate restriction conditions of the IPSA plan according to the reasonable standard of the clinical situation of patients. Attention must also be paid to ensure the dose of HRCTV D90 and improve the dose and suitability on target area and avoid insufficient dose in important target area. Using IPSA needs to notice the occurrence of dose loss in HRCTV, especially in the cervical region and make the dose distribution more reasonable and suitable for cervical cancer treatment.

## Figures and Tables

**Table 1:** General characteristics of patients.

Patients	Average	Median
Age (mean ± SD)	55.26 ± 7.46	54.66
Pathological type	Number	Percentage (%)
Squamous cancer	28	93.33
Adenocarcinoma	2	6.67
Stages		
IIA1	3	10
IIA2	3	10
IIB	5	16.67
IIIA	1	3.33
IIIB	1	3.33
IIIC1	12	40
IIIC2	1	3.33
IVA	2	6.67
IVB	2	6.67
Histologic grade		
Well differentiated	0	0
Moderately differentiated	14	46.67
Poorly differentiated	2	6.67
Unspecified	14	46.66

**Table 2:** Group one (routine condition).

ROI	Usage	Margin (cm)		Surface				Volume			
		Dose	Activity	Min (cGy)	Weight	Max (cGy)	Weight	Min (cGy)	Weight	Max (cGy)	Weight
HRCTV	Ref. target	0	0	600	100						
Bladder	Organ	0	0			500	80				
Rectum	Organ	0	0			370	80				
Sigmoid	Organ	0	0			400	80				

ROI, region of interest; Ref. target, reference target.

**Table 3:** Group two (optimized condition).

ROI	Usage	Margin (cm)		Surface				Volume			
		Dose	Activity	Min (cGy)	Weight	Max (cGy)	Weight	Min (cGy)	Weight	Max (cGy)	Weight
HRCTV	Ref. target	0	0	600	100	600	100	600	100		
Bladder	Organ	0	0			500	80				
Rectum	Organ	0	0			370	80				
Sigmoid	Organ	0	0			400	80				

ROI, region of interest; Ref. target, reference target.

**Table 4:** Group four (optimized condition + target optimization).

ROI	Usage	Margin (cm)		Surface				Volume			
		Dose	Activity	Min (cGy)	Weight	Max (cGy)	Weight	Min (cGy)	Weight	Max (cGy)	Weight
HRCTV	Ref. target	0	0	600	95	600	95	600	95		
UHRCTV	Target	0	0	600	100	700	95	600	100		
Bladder	Organ	0	0			500	80				
Rectum	Organ	0	0			370	80				
Sigmoid	Organ	0	0			400	80				

ROI, region of interest; Ref. target, reference target.

**Table 5:** Radiotherapy mean doses in the four groups.

	Number	HRCTV (cGy, mean ± SD)		D2cc (cGy, mean ± SD)		
		D90	D95	Rectum	Bladder	Sigmoid
Group one	30	607.32 ± 37.86	558.19 ± 38.51	351.49 ± 32.90	423.59 ± 31.39	282.96 ± 44.84
Group two	30	599.01 ± 23.62	558.17 ± 25.72	361.49 ± 28.09	380.75 ± 37.25	273.14 ± 60.69
Group three	30	598.67 ± 13.07	557.03 ± 16.12	370.82 ± 24.44	383.27 ± 32.55	268.94 ± 62.32
Group four	30	596.45 ± 10.94	555.26 ± 12.78	375.33 ± 30.90	385.22 ± 25.79	292.69 ± 52.44

## Data Availability

The data used to support the findings of this study are saved on the main server of brachytheraphy computer in Guangzhou Medical University.
